# Perceptions of tick-borne encephalitis risk: a survey of travellers and travel clinics from Canada, Germany, Sweden and the UK

**DOI:** 10.1093/jtm/tay063

**Published:** 2018-11-22

**Authors:** Cinzia Marano, Melissa Moodley, Elaine Melander, Laurence De Moerlooze, Hans D Nothdurft

**Affiliations:** 1 GSK, Wavre, Belgium; 2 Ipsos MORI, Ipsos Healthcare, London, UK; 3 Department of Infectious Diseases and Tropical Medicine, Medical Center of the University of Munich, Munich, Germany

**Keywords:** TBE, endemic, vaccination, traveller, travel clinic

## Abstract

**Background:**

While the worldwide endemicity of tick-borne encephalitis (TBE) has been increasing, a lack of awareness of the risks of this life-threatening disease may be leading to an underutilization of preventive measures among travellers to TBE-endemic regions. This study’s objectives were to assess travellers’ awareness of TBE and advice-seeking attitudes, and to evaluate practices of travel clinics regarding pre-travel advice.

**Methods:**

We used an online questionnaire to identify individuals aged 18–65 years residing in the UK, Germany, Canada and Sweden, who had travelled to TBE-endemic countries between 2013 and 2016. This sample was defined as the visit-risk sample. Of these, the first 375 respondents who reported that they had engaged in pre-defined at-risk activities (e.g. hiking in forests) were asked to complete an additional online survey and were included in the activity-risk sub-sample. We also used an online/phone questionnaire to interview travel clinic personnel.

**Results:**

The TBE visit-risk sample included 4375 individuals; 69% had heard of the disease and 32% had heard of a TBE vaccine. Before travelling, travellers most commonly sought information online (26%); fewer travellers consulted family doctors (8%) or travel clinics (5%). In the activity-risk sample, 79% of the travellers were aware of at least one correct TBE prevention measure; however, only 15% reported being vaccinated within the past 3 years, with 11% of vaccinated travellers doing so following a clinic’s recommendation. One hundred and eighty travel clinic representatives responded and reported that TBE vaccination was recommended to an average of 61% of travellers to endemic regions. Vaccination-reminder services such as follow-up appointments, e-mail and text reminders were offered by 50% of the clinics.

**Conclusions:**

There is a need to increase awareness of the risk and prevention of TBE among travellers to endemic countries, and travel clinics could play an important role in this process.

## Background

Tick-borne encephalitis (TBE) is a viral infection caused by the TBE virus (TBEV) of the family *Flaviviridae*; three of the TBEV subtypes are known to cause disease in humans.^1,2^ The virus is predominantly transmitted through the bite of an infected tick or, in rare cases, through consumption of unpasteurized dairy products.^2^

The majority of infected individuals remain asymptomatic after the bite, but about 10% of cases develop central nervous system involvement, such as meningitis, encephalitis or myelitis.^2–5^ Case–fatality ratios vary from 0.52% for infections caused by European strains, to up to 35% for the Far Eastern TBE virus subtype.^3^

TBE is endemic across large regions of Europe (in regions of more than 27 countries), Russia and parts of Asia.^2^ The incidence of the disease varies considerably between and even within close geographical regions.^6–9^ In Europe, 1500–3500 TBE cases are reported each year,^10,11^ although this figure is probably underestimated, as disease notification is not mandatory in all countries.^10^ Most cases occur between April and November, with peaks in the summer associated with intense feeding periods during stages of tick development.^2,12^

There is no specific treatment for TBE; however, the disease is vaccine preventable.^13,14^ The World Health Organization recommends vaccination against TBE for people living in risk areas, individuals with occupational risks and travellers to endemic areas, particularly if their visits include outdoor activities.^1^

The incidence of TBE in many countries/geographical areas appears to have increased over the past 30 years.^15^ An overall escalation in international tourist arrivals has also been reported, with Europe experiencing a 5% increase in visiting tourists.^16^ However, travellers are generally unaware of the disease and the majority do not seek any pre-travel advice, or comply with current recommendations when travelling to TBE-endemic regions.^12,15^

In this study, we assessed travellers’ advice-seeking attitudes and awareness of TBE and prevention measures. We also attempted to evaluate practices of travel clinics regarding pre-travel advice.

## Methods

We conducted a questionnaire survey between October and November 2016, which enrolled participants from Canada, Germany, Sweden and UK. The full survey included travellers to TBE- or rabies-endemic regions. Here, we provide the results of the TBE survey. The study methodology is detailed in Supplement 1.

### Traveller survey

We invited respondents aged 18–65 years who had travelled to rabies or TBE-endemic countries in the previous 3 years (visit-risk sample) to complete a 5-min questionnaire. The questionnaire consisted of closed-end questions on travel destinations, preparation for trips and activities, familiarity with travel clinics, awareness and level of concern over TBE and knowledge of preventive measures, including vaccines against TBE. Among the 375 first respondents, a subset of the visit-risk sample who had engaged in high-risk activities completed an additional 15-min survey (activity-risk sub-sample). We considered high-risk activities to be trips that took place between April and November and involved hiking or camping in forests, or cycling/mountain biking. Respondents previously vaccinated against TBE solely due to their residency in a TBE-endemic region were excluded from the survey. The activity-risk questionnaire included both open- and closed-end questions on the perceived risk of TBE and advice-seeking attitudes, as well as knowledge and practice of preventive measures.

### Travel clinic survey

We also surveyed healthcare providers (HCPs; nurses or doctors) working in travel clinics. A screening questionnaire was used to assess their eligibility, with the following inclusion criteria: at least 3 years’ experience of working in a travel clinic, a minimum of 10 h per week spent working in a travel clinic (10 h per month in Sweden), and responsibility for advising on and making decisions about travel vaccination. The questionnaire contained closed- and open-ended questions exploring travel clinics’ practices on pre-travel advice, including risk assessment and prevention recommendations.

The target sample size for the visit-risk survey was >4000 individuals (1000 in each country), and a quota of 375 individuals was pre-defined for the activity-risk survey.

The HCP and traveller data were analysed separately.

## Results

### Visit-risk sample

**Figure 1.**
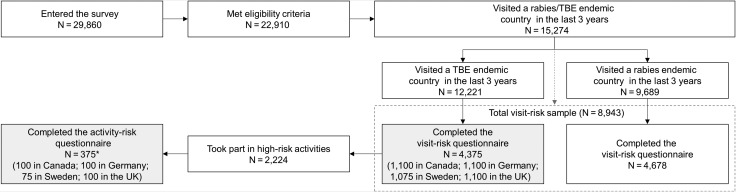
Flow diagram of screened individuals and respondents. TBE, tick-borne encephalitis; *N*, number of respondents; UK, United Kingdom. Note: *the first 375 respondents who had visited a TBE-endemic country took part in high-risk activities and had completed the visit-risk questionnaire were invited to complete the activity-risk questionnaire.

Of the 29 860 screened respondents, 22 910 (77%) met the initial inclusion criteria and 8943 (39%; total visit-risk sample) had visited a rabies or TBE-endemic country and completed the visit-risk questionnaire. Among them, 4375 respondents had visited a TBE-endemic country and were included in the TBE visit-risk sample (Figure 1); a summary of their characteristics is shown in Supplement 2.

The five most frequent TBE-endemic destinations were Germany (visited by 37% of travellers), France (34%), Italy (24%), Denmark (19%) and Greece (17%). The majority of trips took place in July and August.

In the visit-risk sample of travellers to TBE-endemic regions (4 375), 26% prepared for the trip by searching for information online. Other sources of information were reported less frequently, including talking to friends and family who had previously visited the same country (9%), talking to a family doctor (8%), pharmacist (5%), or a travel (5%) or other type (4%)of clinic. Only 14% of travellers to TBE-endemic countries were aware that travel clinics provided travel vaccines, and 52% of respondents were unaware of the existence of travel clinics. Regarding awareness, 24% said they knew a few basic facts about TBE, 10% said they had a good knowledge of TBE, 17% had some understanding and 31% had never heard of the disease. Only a third of TBE visit-risk travellers (32%) were aware of a TBE vaccine, with awareness being higher in Sweden (70%) and lower in Canada (10%) and the UK (12%).

### Activity-risk sub-sample

The TBE activity-risk sub-sample included 375 travellers who completed the activity-risk questionnaire (Figure 1). The average age of the respondents was 41 years; 57% were male and 63% were frequent travellers (Table 1).

**Table 1.** Characteristics of respondents to the activity-risk questionnaire (*N* = 375)

	Canada	Germany	Sweden	UK	Total
Mean age, years	43	41	40	41	41
Age group, %					
18–25 years	8	14	17	8	11
26–35 years	24	22	21	32	25
36–45 years	36	22	25	28	28
46–55 years	12	32	23	15	20
56–65 years	20	10	13	17	15
Male, %	62	55	55	56	57
Travel habits, %					
Frequent traveller	44	69	88	58	63
Occasional traveller	34	23	11	29	25
Infrequent traveller	22	8	1	13	12
Highest level of formal education, %					
Grade school or some high school		7	4	3	3
Completed high school	7	20	35	14	18
Technical or trade school / community college	8	9	8	16	10
Community college or university, but did not finish	10	25	9	8	13
Complete university degree (e.g. Bachelor’s)	44	25	35	43	37
Post-graduate degree (e.g. Master’s or PhD)	31	14	9	16	18
Employment status, %					
Employed full-time	76	67	69	68	70
Employed part-time	13	15	15	22	16
Not employed	3	12	13	5	8
Retired	8	6	3	5	6

*N*, number of respondents; %, percentage of respondents in each category; UK, United Kingdom.

**Figure 2.**
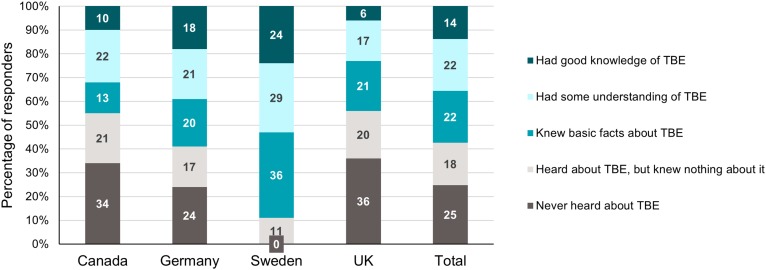
Awareness of TBE among travellers in the activity-risk sub-sample. TBE, tick-borne encephalitis; UK, United Kingdom

Overall, a quarter of the respondents were not aware of TBE at all, although this percentage varied by country, ranging from 36% and 34% in the UK and Canada, respectively, down to 0% in Sweden (Figure 2).

Considering all the trips they had taken to TBE-endemic countries in the past three years, only 14% of the visit-risk sample had ever felt at high risk of TBE on at least one trip and 26% had never felt at risk on any of their trips. Swedes (21%) and Germans (20%) were more likely to have felt at risk, and this percentage was higher among TBE-vaccinated respondents (36%). The perception of risk of TBE was similar among travellers to Europe or Asia (Table 2).

**Table 2.** Perception of risk of TBE (activity-risk sub-sample, *N* = 375)

	% of travellers who felt at risk on at least 1 trip					% of trips to regions where travellers felt at risk	
	Canada	Germany	Sweden	UK	Total	Asia	Europe
Extremely high risk	2	5	1	7	4	2	1
High risk	5	18	21	8	13	2	5
Some risk	16	40	51	21	31	16	16
Very slight risk	40	47	75	47	51	29	28
No risk at all	54	47	55	60	54	39	38
Never felt at risk*	35	21	5	36	26		

TBE, tick-borne encephalitis; *N*, number of respondents; %, percentage of respondents in each category; UK, United Kingdom.

Note: *in any of the trips taken to a TBE-endemic country in the past three years.

In the activity-risk sub-sample, 79% identified at least one correct TBE prevention measure, with the majority being aware of the need to wear long trousers (66%), or to avoid walking in long grass (62%). Only 42% correctly identified vaccination as a prevention measure. Awareness was higher in Sweden, with 100% of travellers identifying at least one correct measure. However, 37% of travellers overall did not take any correct steps to reduce the risk of being infected with TBE (Table 3). June to August were perceived as the months where the risk of contracting the disease is the highest (51–54% of respondents), with travellers from Sweden being more aware of the high-risk period.

**Table 3.** Level of awareness and measures taken to reduce risk of catching TBE by activity-risk travellers (*N* = 375)

	% of respondents	
Measure	Aware of the measure	Taking the measure
Correct		
Wearing long trousers	66	49
Avoiding long grass/sticking to cleared paths	62	Not answered
Tucking trousers into socks	53	28
Being vaccinated against TBE	42	23
Using insect repellent	40	38
Avoiding contact with livestock	27	31
Travelling to risk areas in periods of lower risk	26	24
Incorrect		
Removing any ticks quickly	62	32
Using antiseptic cream on tick bites	24	19

TBE, tick-borne encephalitis; *N*, number of respondents; %, percentage of respondents in each category.

Approximately one-third (35%) of the activity-risk travellers had prior awareness of the TBE vaccine, with 76% of respondents from Sweden and 12% of respondents from the UK being aware of it. Awareness was more common among women (42% vs. 31%) and frequent travellers (43% vs. 23% for occasional or infrequent travellers).

Among the activity-risk travellers, 15% had been vaccinated against TBE, with higher levels observed among Swedes (24%), Germans (21%) and frequent travellers (17%). Reasons cited by travellers for being vaccinated included: knowing they would spend time outdoors (42%), recommendation from their doctor (38%), or friends and family (36%), or for their own peace of mind (22%). Only 11% of respondents had been vaccinated because it was recommended by a travel clinic. Among the vaccinated travellers, 60% stated that the need to have all vaccine doses was made clear to them. However, of those who remembered the number, 50% and 34% reported to have received three or two doses, respectively. Most travellers received the TBE vaccination at their family doctor’s office (53%) or at a travel or vaccination clinic (16%).

### Healthcare provider sample

A total of 180 travel clinic respondents were included in the survey (47 from Canada, 33 from Sweden and 50 from each Germany and the UK). Respondents were doctors (62%) or nurses (38%), with an average 12 years in practice.

When assessing the risks of TBE associated with travel, 76% of HCPs inquired about the travellers planned activities and 76% about the TBE-endemicity of the travel area, while only 23% of HCPs questioned the traveller on the season and 17% on the length of the travel. Only 58% of HCPs agreed that TBE vaccination should be considered as a travel vaccine for those going to endemic countries. Travel clinics recommended TBE vaccination to 61% of travellers to TBE-endemic countries, ranging from 47% in Sweden to 78% in Germany.

The main reasons for recommending TBE vaccination to travellers were planned outdoor activities (for 75% of HCPs), travel to rural areas (58%), multiple destinations (54%), length of trip (48%) and travel to remote areas (46%). HCPs most commonly supported their recommendation by giving information about the risks and complications of TBE (30%), or the seriousness of the disease (22%); 21% also mention the benefits of vaccination and 76% offer advice on prevention measures.

In our study, travel clinics considered that 81% of travellers generally complete a recommended vaccination schedule, and use several types of reminders. Among TBE-vaccinated travellers, the most reported reminders were vaccination cards (for 49% travellers), scheduled follow-up appointments (47%) or vaccination wallet reminder cards (44%) (Table 4).

**Table 4.** Vaccination-reminder services offered by travel clinics and their use among activity-risk travellers

Reminder	Travellers vaccinated against TBE			Travel clinics
	Offered, used	Offered, not used	Not offered	Offered
Written on vaccination card/ booklet	49	18	22	not answered
Follow-up appointment (scheduled at 1st injection)	47	5	31	50
Vaccination wallet reminder card	44	13	31	37
Reminder phone calls	20	15	51	35
A web key tool*	18	11	58	Not answered
An e-mail/ text	18	20	45	38
A website (requiring registration)	9	16	58	10
A vaccine app for smartphone	9	13	64	8

TBE, tick-borne encephalitis.

Note: *A plug-in tool which linked the traveller to a reminder website, provided by a pharmacy.

The HCPs and the travellers gave different weight to reasons for not getting vaccinated, although the main reason was considered the lack of a high risk by both HCPs and travellers (Table 5).

**Table 5.** Main reasons for not taking TBE vaccination, from the travellers and HCPs’ perspective

Travellers (activity-risk sub-sample)				Travel clinics	
	All, %	Visited a HCP before travel, %			
Reason	*N* = 320	Yes (*N* = 94)	No (*N* = 226)	Reason	%
TBE risk was not high enough to need vaccination	34	29	37	Travellers do not consider TBE risk high enough	60
Never really thought about it	27	16	31	Travellers have been to the same country before	48
Nobody told them to get vaccinated	23	16	25	Travellers did not have enough time	44
Did not have enough information	15	14	16	Vaccination safety/side-effects concerns	42
Lack of time/logistical burden	11	23	6	Vaccine is too expensive	39
Had been to the same location before	10	9	10	Travellers do not like needles	38
Uncertainty on whether the vaccine is effective	10	14	8	Vaccine schedule is too arduous	34
Their doctor/nurse/pharmacist did not suggest it	9	14	8	Vaccination takes too much time	32
Did not find out about it until after their travel	9	15	6	Travellers are not sure if vaccine is effective	28
Cost burden	8	15	5	Vaccination cost not reimbursed	23
Their doctor/nurse/pharmacist said it was not needed	6	10	5	Travellers do not feel sufficiently informed on the vaccine	17

TBE, tick-borne encephalitis; HCP, healthcare provider; *N*, number of respondents; %, percentage of respondents in each category.

## Discussion

This questionnaire-based study is one of the first to assess the perception of international travellers and travel clinic HCPs on TBE, a neglected disease despite the known health risks in common tourist destinations in Europe and Asia.

In our study, the profile of the activity-risk sub-sample was based on the at-risk traveller population, for which the World Health Organization currently recommends vaccination against TBE before travelling.^1,4,17^ Although current TBE vaccines provide good protection, and are generally well-tolerated,^14^ only 35% of the activity-risk sub-sample were aware of a vaccine, and only 15% reported being vaccinated. Vaccine uptake for TBE vaccine appears to be low compared with other travel vaccines against infectious diseases. For example, in a sample of travellers similar (for hepatitis A and B) to the visit-risk travellers in this study, 72% were aware of vaccines for hepatitis A and B.^18^ However, in the present study, only 32% of the visit-risk sample were aware of a vaccine for TBE, and this was mainly driven by increased awareness from countries with TBE-endemic regions (Germany and Sweden). Moreover, among the HCPs, only 58% agreed that TBE vaccination should be considered as a travel vaccine for those going to endemic countries/regions. For travellers in the activity-risk sub-sample who were vaccinated, adherence to the vaccination schedule observed was also low, with only half of those who could remember, completing a three-dose series and around a third receiving two doses.

In our study, 79% of the activity-risk sub-sample reported being aware of at least one measure against TBE, which is higher than previous reports of travellers in Canada,^19^ and the UK.^20^ This suggests that the activity-risk travellers in our study were better informed about the prevention of TBE compared with general travellers. However, despite a fairly high awareness, 37% of activity-risk travellers did not take any prevention measure.

From a HCP’s perspective, TBE vaccination was recommended to 61% of travellers. Both HCPs and travellers reported that the main reason for refusing vaccination was the perception that the risk of TBE was not high enough. This was also the most important reason given for declining immunization in Sweden, as reported in a recent study in 8 000 individuals living in a region where vaccination is recommended by local authorities.^21^ Travellers often cited lack of information as a reason for not being vaccinated, while HCPs did not consider this was an important factor in following recommendations. Consistent with previous studies,^12,15^ the HCPs in our survey may have underestimated the incidence of TBE, so it is important that they keep up-to-date with recommendations on TBE to travellers to endemic countries. Vaccination side-effects were perceived as an important reason for vaccine refusal from the travel clinic perspective but not by travellers. In addition, although TBE cases occur between April and November, only 23% and 17% of HCPs included the season and length of trip, respectively, in their top three important components of a TBE risk assessment.

Adherence to the three-dose schedule in our study was 50% based on travellers’ recall. To improve compliance with the vaccination schedule, follow-up appointments and vaccination cards were used more often by travellers than emails, texts or phone calls. This finding was in-line with observations in the rabies sample (Marano *et al.* published in this issue) as well as in a previous study that assessed the use of reminders in travellers vaccinated against hepatitis.^18^ However, although completion of a series of vaccinations (two or three doses) is needed by at least 3 weeks before exposure,^14,22^ only about half of travel clinics offered reminder services.

Understanding of the relationship between travel and vaccination is limited because previous studies have tended to sample from travel clinics or airports, which would reflect travellers rather than the general public.^16,23–27^ Airport surveys may over-represent frequent travellers and are also limited to air travellers, not necessarily capturing inter-Europe travel to TBE-endemic areas, which may also be via rail, car or water. A key strength of our methodology is that it captures travellers to endemic countries, including frequent travellers. However, selection bias is inherent in a self-selected sample, and the online format of the questionnaire meant that the population was limited to those with Internet access. Selection bias was also a limitation of the travel clinic survey. A further limitation was that feedback during the recruitment phase with travel clinic respondents suggested that the concept of ‘travel clinics’ does not exist in Sweden, rather travellers are more likely to attend vaccination sessions at their local health centre.

## Conclusions

Knowledge of TBE varied across the countries studied, with a clear link to TBE-endemicity and travellers from Canada and UK having the lowest awareness. Seeking online information was a common step in travel preparation, suggesting that improving online resources about TBE might increase awareness among travellers. However, for travellers who were vaccinated, many did not complete the vaccination course, suggesting that better vaccination-reminder systems are needed. In addition, more could be done to offer travel clinics better evidence-based tools to help communicate the need for vaccination, including the increased risk of exposure to TBE during summer peaks in endemic countries.

## Supplementary Material

Supplementary Data

Supplementary Data

## Previous presentations in conferences/congresses

Cinzia Marano, Melissa Moodley, Laurence De Moerlooze, Hans Nothdurft. *Risky Travel? Travellers’ Awareness of Rabies and Tick-borne Encephalitis and Factors Influencing Their Health Choices.* Poster presentation, 15th conference of the International Society of Travel Medicine (CISTM15), Barcelona, Spain, 14–18 May 2017.
