# Supplementary material for: Perceptions of tick-borne encephalitis risk: a survey of travellers and travel clinics from Canada, Germany, Sweden and the UK
Source: J Travel Med. 2018 Nov 22;26(Suppl 1):S10–6. doi: 10.1093/jtm/tay063 (PMC6377183; doi:10.1093/jtm/tay063)
Supplement: Supplementary Data [file tay063_tram1501_joint_supplements_final.docx]

# Supplement 1. Methods

This questionnaire-based study was conducted in Canada, Germany, Sweden and United Kingdom [UK] to assess the perceptions of the risk and health choices for rabies and tick-born encephalitis (TBE) among travellers and healthcare providers (HCPs) in travel clinics. The survey was conducted between October and November 2016, and for rabies and TBE, there were two surveys: a max 20-minute survey with travellers to endemic countries, and a 25-minute survey with nurses or doctors working in a travel clinic.

## Recruitment and eligibility criteria

The online panels consisted of pre-recruited individuals who had previously agreed to participate in online research surveys. An invitation for the study was sent via e-mail and eligibility was assessed using a screening questionnaire. In addition to criteria based travel to a TBE and/or rabies-endemic country or region in the past three years. Individuals who had been vaccinated against TBE exclusively because they lived in an endemic region were excluded from the survey. Inclusion criteria were:

- aged 18–65 years
- residence in one of the four countries: Canada, Germany, Sweden, and United Kingdom (UK)
- had travelled to a rabies or TBE endemic country or region in the past three years.

All respondents meeting these criteria were included in the visit-risk sample.

A separate sub-sample (activity-risk sub-sample) was also recruited to answer further questions, based on the following additional criteria:

For the rabies activity-risk sample:

- trip lasted four (4) weeks or longer, OR
- part or all of the trip spent far away from any hospitals or medical centers, OR
- contact with animals during trip.

For the TBE activity-risk sample:

- trip took place between April and November, AND
- hiking or camping in forest or jungle, OR
- cycling or mountain biking.

Respondents for the travel clinics’ survey were identified by contacting the clinics by email, telephone or visits. Recruitment methods are given in Table A.

**Table A**. Recruitment methods for travel clinic respondents

| Canada, Germany, Sweden |  | **UK** |
| --- | --- | --- |
| Initial recruitment efforts via agency’s existing contact list  As recruitment proved challenging this way, efforts were expanded to include personal visits to travel clinics as well as phone calls |  | Email invitation sent to HCP panel  Travel clinics contacted by email or telephone  Respondents completing interviews asked to provide referrals |
| All respondents had the option of completing the survey online or by phone |  | Those contacted by telephone given option to complete interview by phone or online |

Eligibility of HCPs was assessed via a screening questionnaire, based on the following inclusion criteria:

- at least three years’ experience of working in a travel clinic;
- a minimum of 10 hours per week spent working in a travel clinic (10 per month in Sweden);
- responsibility for advising on, and making decisions about, travel vaccination;
- had administered at least five (for UK and Germany) or three (for Sweden and Canada) rabies vaccines a month.

In the UK, Germany and Sweden, travel clinic respondents had to come from centers offering both the rabies and TBE vaccine. The same sample group answered questions on both conditions. In Canada, due to difficulties finding sufficient respondents from centers offering TBE vaccination, this was relaxed to allow a proportion to come from clinics offering the rabies vaccine only.

## Questionnaires

The visit-risk questionnaire was used for both rabies and TBE respondents with separate quotas applied for each group: rabies (850) and TBE (375). Where relevant, these have been analysed together as a total sample (e.g. questions on travel destinations, awareness of conditions, familiarity with travel clinics etc.). Other questions relating specifically to trip are analysed by rabies or TBE visit-risk sample separately. The visit-risk survey consisted of closed questions only.

The travellers’ activity-risk questionnaire was specific to the relevant condition and contained closed and open-ended questions. It aimed to understand perceived levels of risk and determine awareness and use of different measures to reduce the risk of rabies/TBE (including vaccination). Certain questions were asked on a trip basis for all relevant trips the respondent had taken in the past three years (capped at a maximum of three trips to the same country).

Rabies vaccinated individuals were asked to report the type of vaccination received [Pre-exposure vaccination (PrEP) or Post-exposure vaccination (PEP)].

Both TBE and rabies vaccinated respondents in the activity risk group also provided information on the main reasons for having the vaccine, where they had received it and the number of doses. Questions regarding reminder services for vaccination were included. Reasons for non-vaccination prior to travel were collected from unvaccinated individuals in the activity risk sample. Demographic data included age, gender, country of residence, household income, and highest level of formal education.

The travel clinics’ questionnaire contained closed and open-ended questions and aimed to understand how risk assessments are undertaken and the circumstances under which a rabies or TBE vaccination would be recommended. Levels of vaccine uptake among travellers, including perceptions of key reasons for refusal were also included, as well as the system of reminders in place at that clinic. Additional questions were also asked on affiliations of that travel clinic, as well as vaccine brand use and company preference.

Of the travel clinic sample, the majority (70–79%) in all countries except the UK, were travel clinics not connected to other companies or organizations, in terms of recommending travellers to a specific clinic. In the UK, 72% have a connection to another organization or company – 42% have a connection with a school or university, 16% with traditional travel agents and 14% with travel advice websites. 10% are linked to private companies.

*Ethics*

To ensure fully informed consent for participation, the Institutional Review Board providing ethical approval of the study requested that respondents be told the name of the sponsoring pharmaceutical company. To avoid potential bias to responses, respondents were not told of the name of the study sponsor at the start of the interview. Following completion of the survey it was revealed that this was a GSK sponsored survey and respondents had the option to withdraw their answers. Ethical approval was provided by Quorum Review in Canada, Germany, Sweden, and the UK

*Statistics*

A sample size of >4000 individuals (1000 in each country) for the visit-risk survey, and one of 850 individuals (250 in Canada, Germany and the UK and 100 in Sweden) for the activity-risk survey were planned. Analyses were descriptive and were performed separately for travellers’ and HCP data. Elements of the analysis were designed before study start, but most analyses were *post-hoc*. A code frame was developed and applied to verbatim responses for each open-ended question.

**Figure A.** Interview flow


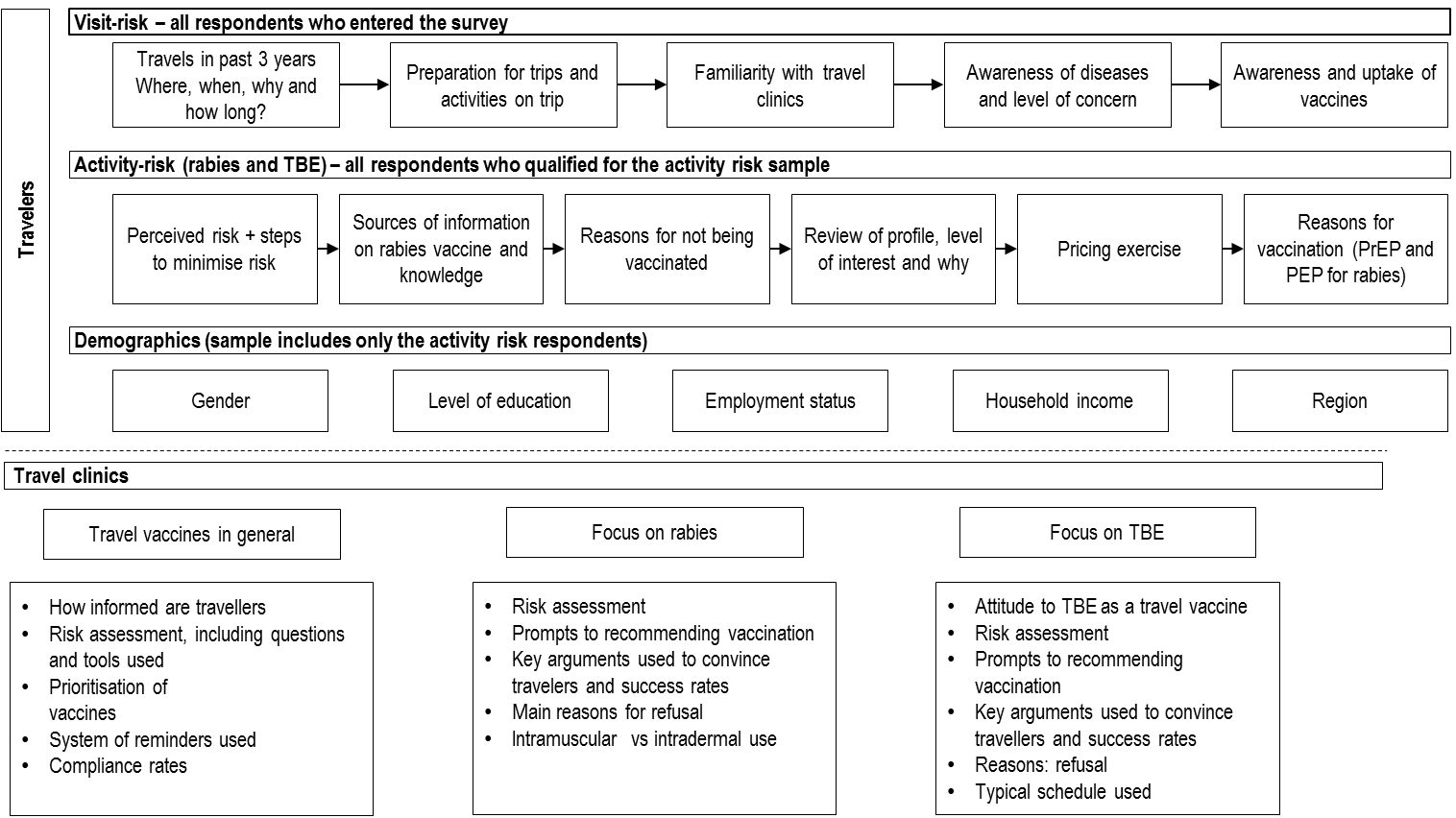


# Supplement 2. Characteristics of travellers in the visit-risk samples

|  |  | Canada | Germany | Sweden | UK | Total |
| --- | --- | --- | --- | --- | --- | --- |
| Total sample (visiting a rabies or TBE endemic country) | | | | | | |
| N |  | 1100 | 1100 | 1075 | 1100 | 4375 |
| Age group, % |  |  |  |  |  |  |
| 18-25 years |  | 5% | 13% | 10% | 5% | 8% |
| 26-35 years |  | 20% | 20% | 24% | 16% | 20% |
| 36-45 years |  | 23% | 22% | 21% | 22% | 22% |
| 46-55 years |  | 26% | 28% | 24% | 26% | 26% |
| 56-65 years |  | 27% | 18% | 20% | 31% | 24% |
| Travel habits, % |  |  |  |  |  |  |
| Frequent traveller |  | 28% | 60% | 66% | 32% | 31% |
| Occasional traveller |  | 38% | 24% | 20% | 37% | 41% |
| Infrequent traveller |  | 37% | 16% | 14% | 31% | 27% |
| Rabies sample (visiting a rabies endemic country) | | | | | | |
| N |  | 1250 | 1250 | 1010 | 1168 | 4678 |
| Age group, % |  |  |  |  |  |  |
| 18-25 years |  | 4% | 13% | 12% | 8% | 9% |
| 26-35 years |  | 19% | 24% | 25% | 26% | 23% |
| 36-45 years |  | 25% | 23% | 20% | 21% | 23% |
| 46-55 years |  | 24% | 23% | 22% | 21% | 23% |
| 56-65 years |  | 27% | 16% | 20% | 24% | 22% |
| Travel habits, % |  |  |  |  |  |  |
| Frequent traveller |  | 29% | 40% | 23% | 25% | 30% |
| Occasional traveller |  | 38% | 32% | 34% | 38% | 36% |
| Infrequent traveller |  | 33% | 28% | 43% | 37% | 35% |

TBE, tick-born encephalitis; N, number of respondents in each category.

# **Supplement 3.** Questions typically asked by travel clinics to conduct a general risk assessment of travellers

|  | Frequency, % |
| --- | --- |
| Trip related |  |
| Length of trip | 84% |
| Travel to specific areas which are endemic for certain diseases | 78% |
| Activities likely to take place on their trip | 76% |
| Travel to urban vs rural areas | 76% |
| Length of time until departure | 67% |
| Season of trip | 66% |
| Availability of medical care | 57% |
| Self-organised vs group organised holiday | 48% |
| Travel insurance | 46% |
| Visiting of friends/family | 44% |
| Patient related |  |
| Past vaccinations | 84% |
| Current medication | 76% |
| Currently pregnant or planning pregnancy | 72% |
| Previous adverse reactions to vaccines | 70% |
| Currently breastfeeding | 59% |
| Person's ability / willingness to pay for the vaccine | 46% |
| Other | 16% |
